# Innovative Strategies for the Targeted Degradation of Viral Proteins: Paving the Way for Next-Generation Therapeutics

**DOI:** 10.3390/pharmaceutics17111420

**Published:** 2025-11-03

**Authors:** Alexander S. Sobolev, Georgii P. Georgiev

**Affiliations:** 1 Faculty of Biology, Lomonosov Moscow State University, 1-12 Leninskiye Gory St., 119234 Moscow, Russia; 2 Institute of Gene Biology, Russian Academy of Sciences, 34/5 Vavilov St., 119334 Moscow, Russia; georgiev@genebiology.ru

**Keywords:** viral proteins, targeted protein degradation, PROTACs, modular nanotransporters, diving antibodies, intracellular delivery

## Abstract

**Background/Objectives**: This review discusses the development and application of targeted protein degradation strategies, particularly focusing on the ubiquitin–proteasome pathway and PROteolysis-TArgeting Chimeras (PROTAC) technology, for antiviral therapies. **Methods/Results**: The synthesis of specific PROTACs exemplifies the potential of this approach to inhibit viral replication. The discussion also covers ongoing efforts to develop broad-spectrum antivirals and explores the limitations of small-molecule ligands, proposing antibody mimetics as a versatile alternative. The review details innovative strategies involving engineered antibody mimetics, termed ‘diving antibodies’ (DAbs), capable of intracellular delivery and targeting viral proteins within cells. These molecules are engineered using modular nanotransporters to facilitate intracellular delivery. The integration of E3 ligase-binding sites into DAbs enhances their capacity to induce targeted protein degradation, with experimental evidence supporting their efficacy. **Conclusions**: Overall, the review underscores the potential of combining targeted degradation technologies with innovative delivery systems to create effective antiviral therapies, especially for viruses with limited treatment options.

## 1. Introduction

Human cells possess an extensive repertoire of ubiquitin ligases and a vast array of deubiquitinating enzymes, ranging from highly specific to broadly acting types. This remarkable diversity offers a powerful toolkit for developing both targeted and general strategies to counter viral infections. Several authors have considered this possibility [1,2], and recently the first experimental studies on this subject emerged.

One of the first experimental publications devoted to the possibility of targeted degradation of viral proteins [3] provided compelling evidence that the tripartite motif protein 14 (TRIM14) plays a significant role in inhibiting hepatitis C virus (HCV) replication. This study demonstrated that TRIM14 facilitated the degradation of the viral non-structural protein NS5A, a critical component for viral replication. This degradation process involves the SPRY domain of TRIM14 and is likely mediated through the K48-linked ubiquitination pathway, which targets proteins for proteasomal degradation. In general, TRIM proteins are a family of E3 ubiquitin ligases involved in many cellular processes [4]. Later, Zhao et al. [5] discovered that the metabolite APL-16-5 exerts its antiviral activity against the influenza A virus by binding the E3 ubiquitin ligase TRIM25, thereby promoting the degradation of the viral polymerase via ubiquitination.

Chatterjee et al. [6] developed peptides targeting the SARS-CoV-2 spike protein’s receptor-binding domain, recruiting E3 ubiquitin ligases to promote viral degradation via the proteasome. These peptides effectively degraded the receptor domain in human cells and inhibited viral production.

## 2. PROTACs

**Figure 1:**
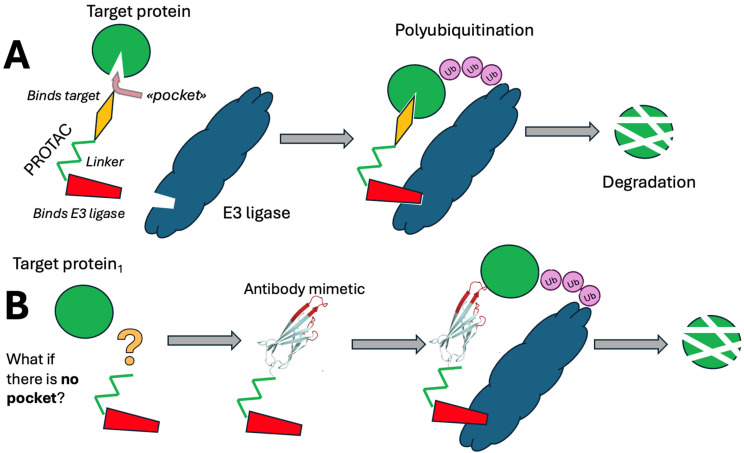
A simplified scheme illustrating the PROTAC-based degradation of a target protein (**A**) and a diagram showing the potential use of an antibody mimetic for binding to a protein lacking a ligand-binding pocket (**B**).

PROTACs are of particular interest because they possess qualities that enable them to be considered as potential drugs [7,8,9,10,11]. The heterobifunctional molecules of PROTACs consist of two ligands connected by a linker. One ligand targets the protein of interest, while the other binds to an E3 ubiquitin ligase, facilitating the transfer of ubiquitin to the protein and leading to its subsequent degradation (Figure 1A).

PROTACs possess several notable advantages over traditional protein inhibitors [8]. Unlike conventional inhibitors that primarily block the activity of a target protein, PROTACs facilitate the degradation of the protein. It results in a more comprehensive and sustained suppression of the protein’s function. One of the key benefits of PROTACs is their catalytic mode of action. A single PROTAC molecule can induce the degradation of multiple copies of the target protein, thereby reducing the required drug dosage. This catalytic property not only enhances the therapeutic efficiency but also minimizes potential off-target effects and toxicity associated with higher drug concentrations. PROTACs also offer a strategic advantage in overcoming drug resistance. Resistance often develops with traditional inhibitors due to mutations in the active site of the target protein. Since PROTACs lead to complete protein degradation rather than mere inhibition, they can bypass resistance mechanisms caused by such mutations, maintaining therapeutic efficacy. Additionally, because PROTACs operate catalytically, they generally require lower doses compared with traditional inhibitors, which contributes to a more favorable therapeutic profile. The unique properties of PROTACs position them as a promising therapeutic strategy for a range of diseases, including cancer and neurodegenerative disorders. According to the clinicaltrials.gov database, approximately two dozen PROTACs have now reached clinical trials, most of which are developed for the treatment of cancer. The creation of PROTACs for the treatment of viral diseases began later than the development of anti-cancer PROTACs. However, this delay can be advantageous, providing an opportunity to address issues identified during clinical trials of PROTACs for cancer treatment [8]. These issues include limited specificity, off-target effects causing toxicity, and suboptimal pharmacokinetics.

Thus, a team from Harvard Medical School and Dana-Farber Cancer Institute synthesized a PROTAC, DGY-08-097, which binds to the HCV NS3/4A protease active site and recruits the E3 ubiquitin ligase complex [12]. This DGY-08-097 causes the degradation of the HCV NS3/4A protease and inhibits HCV replication in cell cultures.

In 2021, a Chinese patent application was published describing a novel PROTAC targeting influenza A virus neuraminidase [13]; it was followed by an article describing this development [14]. The PROTAC contained the anti-influenza drug oseltamivir, which recognized the target protein. The authors investigated 25 variants of PROTACs with N-substituted oseltamivir, varying the types and lengths of the linkers as well as the ligands to different E3 ligases. They assessed the anti-H1N1 activity using EC_50_ values obtained from plaque formation assays in MDCK cells. The most effective variant was 8e, which featured a nine-carbon alkyl chain and a ligand to Von Hippel-Lindau E3 ligase (VHL), demonstrating the highest antiviral activity with an EC_50_ of approximately 0.33 μM. It induced the degradation of neuraminidase and inhibited influenza A virus replication. Later, another group [15] developed a pentacyclic triterpenoid PROTAC, consisting of an oleanolic acid derivative and a linker to VHL, which induced the degradation of the hemagglutinin of the influenza A virus. This compound functioned through a ubiquitin- and proteasome-dependent mechanism and exhibited activity against the influenza A virus. The PROTAC effectively protected mice from the toxic effects induced by influenza A virus infection.

In the same year, the German Federal Agency for Disruptive Innovation (SPRIND) initiated an innovation competition to identify and fund strategies for platform-based broad-spectrum antivirals. The objective of this challenge was to establish a foundation for the development, testing, production, and distribution of antiviral medications in advance of future pandemics, ensuring the availability of effective countermeasures from the outset of such events. The progress report on the SPRIND Challenge “Broad-Spectrum Antivirals” (December 2023) [16] presented six approaches that received funding, and PROTACs were among them. The report mentions—without any detail—the work of the PROTACs team, which led to the development of PROTACs against SARS-CoV-2 (the virus that causes COVID-19), which are currently in the lead optimization phase for preclinical development. This team has more recently produced MproPROTAC that targets the SARS-CoV-2 main protease, Mpro. Mpro is a highly conserved enzyme across various coronaviruses, playing a crucial role in viral replication and pathogenesis. The authors characterized the ternary complexes of Mpro, MproPROTAC, and Cereblon, a component of the E3 ubiquitin ligase complex [17]. However, before this team, a group from Texas A&M University [18] achieved results with MPD2, their PROTAC targeted at Mpro, which contained an Mpro inhibitor, MPI8, and a Cereblon ligand. MPD2 effectively decreased MPro levels in SARS-CoV-2-infected A549-ACE2 cells. The compound also exhibited broad antiviral activity against multiple SARS-CoV-2 strains, including those resistant to nirmatrelvir, a commonly used antiviral drug. Another group [19] modified the clinically approved Mpro inhibitor nirmatrelvir into a series of degraders. Three compounds derived from nirmatrelvir and utilizing E3 ligase recruiters effectively degraded Mpro. The most potent compound showed enhanced antiviral activity against multiple wild-type and resistant strains compared with the non-degrading controls.

In 2021, Hahn et al. [20] developed THAL-SNS032, a cyclin-dependent kinase (CDK)-inhibitor-based PROTAC with activity against human cytomegalovirus. This compound demonstrated a verified degradative mechanism and showed a fourfold increase in anti-viral activity compared with its parent inhibitor, along with a broader anti-viral spectrum.

A recent paper [21] described a PROTAC molecule targeted at the SARS-CoV-2 3-chymotrypsin-like protease. This molecule combined a ligand for the protease with pomalidomide, which attracts E3 ligase. The PROTAC caused an efficient decrease in the protease level in HeLa cells expressing the protein. Another PROTAC, L15, which targeted the viral infectivity factor (Vif) of human immunodeficiency virus-1 (HIV-1), degraded the Vif protein in a dose-dependent manner [22]. Table 1 summarizes the papers on the creation of PROTACs aimed at combating viral infections.

An approach similar to those described above was used for the targeted degradation of E6 and E7 oncoproteins of the human papillomavirus for the treatment of head and neck cancer [23]. The same authors proposed the use of exosomes to deliver PROTACs aimed at the degradation of proteins of H3N2 influenza A virus and human immunodeficiency virus [24,25].

## 3. Diving Antibodies

The PROTAC approach offers several notable advantages, most prominently the capacity to attain a sufficiently high specificity for the target protein [8]. However, PROTAC strategies face limitations primarily due to their reliance on engineered small-molecule ligands, typically inhibitors, to target specific proteins. These ligands are generally effective for enzymes, ion channels, and receptors with accessible binding pockets. However, for proteins lacking such pockets, the development of suitable low-molecular-weight ligands becomes challenging, restricting the applicability of the PROTAC technology. Consequently, this hampers the creation of a universal approach for targeted protein degradation using classical PROTACs. In contrast, antibodies and antibody-like molecules, i.e., antibody mimetics, offer broader versatility, as they can be generated against a wide range of antigens, including those inaccessible to small-molecule inhibitors. These biological tools can be tailored to recognize various post-translational modifications, splicing variants, and isoforms, making them more adaptable for targeted protein regulation in diverse biological contexts. It is apparent that the integration of highly specific antibody mimetics could substantially facilitate the resolution of this issue, provided that these antibody mimetics possess the requisite ability to penetrate living cells and access the desired intracellular compartments. Such advancements (Figure 1B) would not only enhance the efficacy of PROTACs but also expand the range of accessible sites within a target molecule.

Recently, Khramtsov et al. [26,27,28,29,30,31] developed a technology for generating antibody mimetics capable of penetrating target cells, termed ‘diving antibodies’ (DAbs). These engineered molecules enter cells via receptor-mediated endocytosis, navigate to defined intracellular compartments, and bind specific intracellular proteins—thus enabling precise modulation of molecular processes. This technology builds upon the principle of modular nanotransporters [26], artificial chimeric proteins composed of functional modules that act sequentially to direct intracellular transport. Each modular nanotransporter contains (1) a ligand module that binds to internalizable receptors on target cells, (2) an endosomolytic module that mediates escape from endocytotic vesicles, (3) a transport module that determines subcellular localization, and (4) a carrier module that maintains structural integrity. In DAbs, an additional module (5)—an antibody mimetic—was incorporated to confer specific binding to the chosen intracellular protein. The selection of the ligand module (1) depends on the repertoire of the internalized receptors of the target cells. In some cases, target cells may lack suitable receptors for effective internalization. In such situations, employing cell-penetrating peptides as part of the DAbs with E3LBP (as a module (1)) can provide internalization of the DAbs. Together, these modules allow DAbs to exploit endogenous cellular transport pathways to reach their destination and selectively interact with target proteins inside the cell.

This approach has already enabled the targeting of various DAbs to different intracellular compartments. For example, Keap-1-specific DAb targets Keap 1 at the outer membrane of mitochondria [27,28,29]; c-Myc-specific DAb translocates to the nucleus to bind this oncoprotein [30], while a DAb specific to the nucleocapsid (N) protein of the SARS-CoV-2 virus [31,32,33,34,35] targets the N protein in the hyaloplasm. The confirmation of successful complex formation between DAbs and their target proteins within living cells was experimentally confirmed by two methods: Fluorescence Lifetime-based Fluorescence Resonance Energy Transfer and Cellular Thermal Shift Assay. Although the technology of DAbs has emerged only in the last three years, it has already demonstrated its applicability in vivo: DAbs, which interact with Keap1 and thereby release the transcription factor Nrf2 from the complex with this inhibitor, have shown a significant protection against acetaminophene-induced oxidative stress in mice [29].

The modular structure of DAbs provides unique possibilities to regulate their targeting and binding properties. In the case of the SARS-CoV-2 N-protein, the DAb contains two antibody mimetic modules, an NC2 monobody, targeting N-protein as module (5), and an affibody against the internalizable epidermal growth factor receptor as module (1). In addition, the molecule contains a hydrolysis site that enables cleavage of the monobody by acidic proteases within endocytotic compartments. This cleavage releases the NC2 monobody enhancing its binding efficiency to the viral N-protein. In contrast, the non-cleaved form exhibits a lower affinity, indicating that the cleavage process plays a crucial role in optimizing the functional performance of the monobody [35].

The use of monobodies and affibodies within the DAbs is not accidental: these antibody mimetics lack disulfide bonds and remain stable and active in the reducing environment within the cytoplasm, making them suitable for intracellular use [36]. Additionally, the monobody used in the N-protein-specific DAbs was capable of the disassembly of biocondensates formed by N-proteins [32], which are generated via liquid–liquid phase separation. Biocondensates, formed by N-proteins due to the presence of internally disordered regions, function as structural scaffolds, facilitating coronavirus replication. Disruption of these structures inhibits SARS-CoV-2 replication, highlighting their potential as targets for antiviral strategies [37]. The control DAbs lacking the monobody module did not affect biocondensates [32]. It is worth mentioning that disordered short linear motifs of viral proteins may be considered for therapeutic interventions [38,39], with monobody-containing DAbs being one of the top candidates for targeting such motifs.

**Figure 2:**
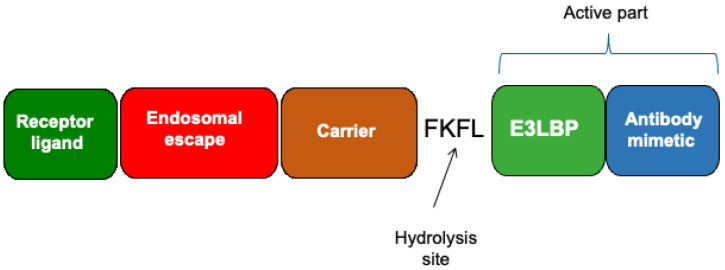
A scheme of DAb with E3LBP designed to degrade the SARS-CoV-2 nucleocapsid protein (after [32]). The DAbs with E3LBP have several functional modules: the receptor ligand module, which employs an affibody targeting internalizable epidermal growth factor receptors; the endosomal escape module, consisting of the diphtheria toxin translocation domain; the carrier module, which is based on the E. coli hemoglobin-like protein; the hydrolysis site, FKFL, which is susceptible to acidic proteases such as cathepsin B; E3LBP, an amino acid sequence that binds to Keap1 E3 ligase; and an antibody mimetic, specifically the NC2 monobody, which targets the SARS-CoV-2 nucleocapsid protein.

The DAb design can be further refined to not only recognize and bind its target but also promote its degradation. This was performed for the design of the N-protein-specific DAb. The step involved integrating the Keap1 E3-ligase-binding peptide (E3LBP) into the DAb [31,32,33]. This site was strategically positioned between the FKFL motif, a hydrolysis site, and the monobody. Such an arrangement ensured the simultaneous cleavage of both the E3LBP and the monobody within the endocytotic compartment (Figure 2). Two cell lines, A549 and A431, were genetically modified to express N-protein. Western blot analysis using N-protein-specific antibodies demonstrated significant degradation of the N-protein in both cell lines upon treatment with DAb. It was possible to calculate the rate of N-protein degradation for these two cell lines, which was approximately 130 nM per hour. To explore the degradation pathways involved, inhibitors targeting proteasomal and autophagic processes were employed. MG132, a proteasome inhibitor, and Bafilomycin A1, an autophagy inhibitor, were selected for this purpose. The results showed that Bafilomycin A1 effectively inhibited N-protein degradation, whereas MG132 did not, indicating that autophagy plays a primary role in N-protein degradation. The monobody used within the DAb with E3LBP to recognize the SARS-CoV-2 N-protein was also capable of interacting with the SARS-CoV N-protein [40]. Therefore, these DAbs with E3LBP may also be effective in treating SARS-CoV coronaviruses. The experiments using DAbs with E3LBP can serve as a model for developing future therapies for viral diseases. The efficacy of this approach will be evaluated in the future using full-sized virus particles.

The growing interest in using antibody mimetics and antibodies for targeted protein degradation is also reflected in the recent review by Hong and An, who highlighted their potential in this emerging field [41]. The authors discuss, among other topics, the development of antibody–drug conjugates aimed at improving the targeted delivery of chemotherapeutic agents. They also considered protein-degrader–antibody conjugates. In these conjugates, the antibody functions to facilitate entry into target cells by binding to the internalizable surface antigens. Once inside the cell, a protein degrader initiates the degradation of the target protein. In other words, the antibody within these conjugates acts as a delivery mechanism rather than a recognition element for the intracellular target protein, which is performed by the antibody mimetic in DAbs with E3LBP. In the context of using antibodies for intracellular protein degradation, it is also important to mention bioPROTACs [42]. These chimeric protein molecules contain, among other components, nanobodies that target specific proteins fused to E3 ubiquitin ligase. For bioPROTACs to induce protein degradation, the cells must be transfected with a plasmid encoding the bioPROTAC. This significantly distinguishes bioPROTACs from DAbs with E3LBP, which can penetrate target cells independently.

A generalized scheme for a molecule designed for the targeted intracellular degradation of viral proteins can be outlined as follows:

- *The Cell Entry Module:* Facilitates entry into cells via receptor-mediated endocytosis or containing cell-penetrating peptides, either specifically or non-specifically.
- *The Endosomal Escape Module:* Enables escape from endocytotic vesicles into the hyaloplasm.
- *The Ubiquitination Module:* Contains an amino acid sequence that binds E3 ubiquitin ligase, promoting ubiquitination of the target protein.
- *The Targeting Module:* Incorporates an antiviral protein-specific antibody mimetic, with an added hydrolysis site by acid proteases between the targeting and ubiquitination modules and the rest of the molecule for controlled degradation within endocytotic compartments.
- *The Subcellular Localization Module* is essential for directing the molecule to specific subcellular compartments beyond the hyaloplasm.

We believe that this modular approach will allow the precise targeting and efficient degradation of viral proteins within host cells, thereby enhancing the therapeutic potential through controlled intracellular delivery and processing.

## 4. Conclusions and Future Perspectives

Developing antiviral agents targeting the ubiquitin-proteasome pathway has attracted significant scientific interest due to its potential to selectively degrade viral proteins and inhibit viral replication. The advent of the PROTAC technology has further advanced this field, offering a versatile platform for targeted protein degradation.

Currently, new and more versatile innovative approaches such as ‘diving antibodies’ have been developed to penetrate cells and specifically target intracellular viral proteins with antibody mimetics. These molecules leverage modular nanotransporters to facilitate cellular entry and intracellular delivery, enabling the degradation of viral components such as the SARS-CoV-2 nucleocapsid protein. The integration of such biological tools with the principles of PROTAC technology could overcome the current limitations related to ligand development, broadening the scope of targeted antiviral therapies. These advancements underscore the potential of combining molecular degradation strategies with innovative delivery systems to develop effective, targeted antiviral treatments capable of addressing current and future viral threats.

## Figures and Tables

**Table 1:** PROTACs used as antivirals and their target proteins.

Virus	Target Protein	PROTAC	Reference
Hepatitis C	NS3/4A protease	DGY-08-097	[12]
Influenza A	Neuraminidase	8e	[13,14]
Influenza A	Hemagglutinin	V3	[15]
SARS-CoV-2	Main protease	MPD2	[18]
SARS-CoV-2	Main protease	BP-198	[19]
SARS-CoV-2	3-chymotrypsin-like protease	PROTAC 1	[21]
Human cytomegalovirus	Cyclin-dependent kinase 9	THAL-SNS032	[20]
Human immunodeficiency virus-1	Vif	L15	[22]

## Data Availability

Data are contained within the article.

## Abbreviations

The following abbreviations are used in this manuscript:

DAb	diving antibody
E3LBP	E3-ligase binding peptide
HCV	hepatitis C virus
Mpro	SARS-CoV-2 main protease
PROTACs	PROteolysis TArgeting Chimeras
TRIM	tripartite motif protein
VHL	von Hippel–Lindau Cullin RING E3 ligase
